# Antioxidant Properties of European Cranberrybush Fruit (*Viburnum opulus* var. *edule*)

**DOI:** 10.3390/molecules15064467

**Published:** 2010-06-23

**Authors:** Otakar Rop, Vojtech Reznicek, Magdalena Valsikova, Tunde Jurikova, Jiri Mlcek, Daniela Kramarova

**Affiliations:** 1 Department of Food Technology and Microbiology, Faculty of Technology, Tomas Bata University in Zlin, Namesti T. G. Masaryka 275, 762 72 Zlin, Czech Republic; E-Mail: mlcek@ft.utb.cz (J.M.); 2 Department of Breeding and Propagation of Horticultural Plants, Faculty of Horticulture, Mendel University in Brno, Valticka 337, 691 44 Lednice, Czech Republic; E-Mail: reznicek@mendelu.cz (V.R.); 3 Department of Vegetables-Production, Horticulture and Landscape Engineering Faculty, Slovak University of Agriculture in Nitra, Tulipanova 7, 949 76 Nitra, Slovakia; E-Mail: magdalena.valsikova@uniag.sk (M.V.); 4 Department of Natural and Informatics Sciences, Faculty of Central European Studies, Constantine the Philosopher University in Nitra, Drazovska 4, 949 74 Nitra, Slovakia; E-Mail: tjurikova@ukf.sk (T.J.); 5 Department of Food Biochemistry and Analysis, Faculty of Technology, Tomas Bata University in Zlin, Namesti T. G. Masaryka 275, 762 72 Zlin, Czech Republic; E-Mail: kramarova@ft.utb.cz (D.K.)

**Keywords:** European cranberrybush, phenolics, antioxidant activity, flavonoids, ascorbic acid

## Abstract

In the literature there is little available information concerning European cranberrybush fruit (*Viburnum opulus* var. *edule*). This plant can be cultivated, even in harsh climatic conditions, because of its low environmental demands, and it is possible to harvest the fruit even in the snow cover. The aim of this study was to determine the content of polyphenolics, antioxidant activity, flavonoids and vitamin C in the fruit of three cultivars ´Leningradskaya otbornaya´, ´Souzga´ and ´Taezny rubiny´ of this species. In the case of polyphenolics, high contents [up to 8.29 g of gallic acid/kg of fresh mass (FM)] were observed. The 1,1´-diphenyl-2-picrylhydrazyl (DPPH) and 2,2´-azinobis-3-ethyl-benzthiazino-6-sulphonic acid (ABTS) tests were applied to determine antioxidant activity, which was also high in comparison with other fruit species. The corresponding correlations between the polyphenolic content and antioxidant activity were in case of the DPPH test r^2^ = 0.88 and for the ABTS test r^2^ = 0.98. For comparison, the scavenging activity towards reactive oxygen species (superoxide anion, hydroxyl radical and nitric oxide) was determined by using a 25% fruit extract of particular cultivars. Antioxidant efficiency was also assessed using the rat liver slice model. Furthermore, the contents of flavonoids and vitamin C were assayed, giving values of 4.89 g/kg and 1.64 g/kg FM, respectively. The work should contribute to the popularization of this species as a promising crop plant in human nutrition.

## 1. Introduction

The species *Viburnum opulus* L. belongs to the *Caprifoliaceae* plant family [1]. The fruits of *Viburnum opulus* var. *edule* (European cranberrybush) and var. *trilobum* Marsh. (American cranberrybush) are edible [2]. While the American cranberrybush is native to North America [3], the plants of European cranberrybush are found in Europe, North Asia and North Africa, and also in the central zone of Russia [4]. The berries of European cranberrybush are astringent, therefore, they are seldom consumed directly, and the fruit juice is the best known product. It is a traditional drink in the Central Anatolia region (Turkey) [5]. Because of its strong astringent taste [4], the fruit flesh can be used in making mixed fruit juices [6]. European cranberrybush fruit is also used locally for preparing jam, jelly, sweetmeat and marmalade. Since the berries have a lot of natural pectins in early autumn (consumption ripeness), added pectins are not needed to produce the products mentioned above [7]. What is also interesting is that during the winter months it is also possible to harvest the fruits because of their resistance to cold weather [8]. Although there is a decrease in the content of bioactive substances, the amounts of dry matter and sugars increase. Therefore, the fruit can become a source of fresh fruit found in nature covered in snow [9]. 

In Russia the berries are used in traditional and folk medicine [7]. The fruit are a source of various biochemical components such as ascorbic acid and flavonoids [10]. The content of phenolic compounds has an influnce on the high antioxidant activity of berries [11]. Sagdic *et al.* [12] noticed antimicrobial activity of methanolic extracts of fruit. Similarly, antimicrobial activity was described in the case of European cranberrybush fruit seed oil [13]. 

Due to the lack of information in the literature, the aim of this work was to determine total phenolic content (TPC), total antioxidant activity (TAA), total flavonoid content (TFC), scavenging activity of reactive oxygen species (ROS) – nitric oxide, superoxide anion and hydroxyl radical, scavenging activity of lipid peroxidation as well as ascorbic acid content (AAC) in *Viburnum opulus* var. *edule* fruit. Three cultivars of European cranberrybush were used, namely ´Leningradskaya otbornaya´, ´Souzga´ and ´Taezny rubiny´ which are all Russian in origin.

## 2. Results and Discussion

**Figure 1:**
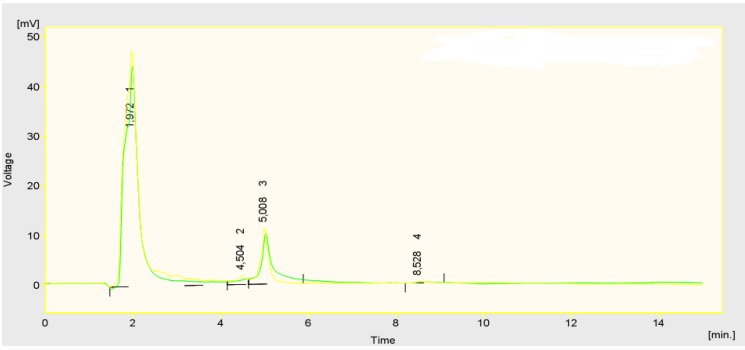
Chromatogram representing the peak of ascorbic acid (retention time 1.872 min.).

The fruit of European cranberrybush are unique because of their composition and a high content of substances having antioxidant activity [6]. In our measurement very high contents of polyphenolics were observed. The values of their contents ranged from 6.80 to 8.29 grams of gallic acid/kg FM. These contents were higher than the values found in European cranberrybush by Akbulut *et al.* [14]. These authors observed 3.25 g of gallic acid/kg FM. For most fruit species lower contents are typical [15]. For example, in apples the common content is between 0.6 to 2.1 grams of gallic acid/kg FM [16] and in plums it is 2.2 to 5.0 grams of gallic acid/kg FM [17]. The results of the measurements performed in the three cultivars showed variability of the other chemical compounds. In case of vitamin C, its contents ranged from 1.01 to 1.64 grams/kg FM. The chromatogram with the marked retention time is shown in Figure 1. In comparison with tabulated data of other fruit species this value is high, nevertheless, it does not reach the values which are observed in, for example, sea-buckthorn (*Hippophae rhamnoides* L.), where an extreme value of 12 grams of ascorbic acid/kg FM can be found [15], as pointed out byErcisli *et al.*, for example [18]. Interestingly, vitamin C was the only factor where statistical significance was demonstrated between the years (see Table 1).

**Table 1:** Total flavonoid and ascorbic acid contents (g/kg FM) of fruits of different cultivars of European cranberrybush (*Viburnum opulus* var. *edule* Marsh.), n = 25.

Cultivar	Year	TFC	AAC
Leningradskaya otbornaya	2007	3.85 ± 0.17^a^	1.14 ± 0.11^a^
	2008	3.96 ± 0.16^a^	1.35 ± 0.09^b^
	2009	4.06 ± 0.20^a^	1.47 ± 0.08^b^
Souzga	2007	3.14 ± 0.21^b^	1.01 ± 0.10^a^
	2008	3.44 ± 0.14^b^	1.12 ± 0.06^a^
	2009	3.25 ± 0.15^b^	1.50 ± 0.10^b^
Taezny rubiny	2007	4.54 ± 0.25^c^	1.64 ± 0.08^b^
	2008	4.75 ± 0.27^c^	1.15 ± 0.11^a^
	2009	4.89 ± 0.21^c^	1.50 ± 0.05^b^

Different superscripts in each column indicate the significant differences in the mean at P < 0.05.

European cranberrybush fruit are a reported to be a good source of flavonoids [19], which was also confirmed in our measurements. The content of flavonoids ranged from 3.14 to 4.89 g/kg FM (Table 2). In other fruit lower values are common [20].

**Table 2:** Scavenging effect of European cranberrybush fruit (*Viburnum opulus* var. *edule* Marsh.) extract (25%) on nitric oxide (percentage of inhibition), superoxide anion (percentage of inhibition), hydroxyl radical (percentage of inhibition) and lipid peroxidation (percentage of inhibition), n = 25.

Cultivar	Year	Nitric oxide (%)	Superoxide anion (%)	Hydroxyl radical (%)	Lipid peroxidation (%)
Leningradskaya otbornaya	2007	24.16 ± 0.26^a^	27.11 ± 0.56^a^	22.02 ± 0.85^a^	12.71 ± 0.56^a^
	2008	23.84 ± 0.32^a^	27.60 ± 0.39^a^	21.86 ± 0.79^a^	12.95 ± 0.29^a^
	2009	24.31 ± 0.29^a^	27.55 ± 0.50^a^	21.90 ± 0.69^a^	13.16 ± 0.28^a^
Souzga	2007	22.11 ± 0.15^b^	25.16 ± 0.49^b^	19.40 ± 0.80^b^	11.20 ± 0.36^b^
	2008	22.10 ± 0.28^b^	25.44 ± 0.50^b^	19.65 ± 0.75^b^	11.62 ± 0.38^b^
	2009	21.89 ± 0.30^c^	25.13 ± 0.45^b^	19.61 ± 0.81^b^	11.51 ± 0.40^b^
Taezny rubiny	2007	25.19 ± 0.31^d^	28.50 ± 0.38^c^	23.94 ± 0.74^c^	13.78 ± 0.51^ac^
	2008	25.44 ± 0.24^d^	27.95 ± 0.30^ac^	23.78 ± 0.68^c^	13.90 ± 0.37^c^
	2009	25.37 ± 0.26^d^	28.15 ± 0.41^c^	23.98 ± 0.80^c^	13.45 ± 0.42^ac^

Different superscripts in each column indicate the significant differences in the mean at P < 0.05.

Considering the fact that all cultivars were grown under identical conditions and in the same locality, it is possible to conclude that one can clearly see cultivar variability, which is quite typical of fruit [21]. This variability became particularly evident in case of total antioxidant activity (see Table 3), where two methods were used for its measurement [the 1,1´-diphenyl-2-picrylhydrazyl (DPPH) and 2,2´-azinobis-3-ethylbenzthiazino-6-sulphonic acid (ABTS) tests]. 

**Table 3:** Total phenolic content (grams of gallic acid/kg FM) and antioxidant activity (grams of AAE/kg FM) of fruits of different cultivars of European cranberrybush (*Viburnum opulus* var. *edule* Marsh.), n = 25.

Cultivar	Year	TPC	TAA (DPPH test)	TAA (ABTS test)
Leningradskaya otbornaya	2007	7.20 ± 0.22^a^	9.21 ± 0.16^a^	9.90 ± 0.14^a^
	2008	7.27 ± 0.15^a^	9.28 ± 0.17^a^	9.95 ± 0.18^a^
	2009	7.29 ± 0.20^a^	9.34 ± 0.10^a^	9.98 ± 0.17^a^
Souzga	2007	6.80 ± 0.15^b^	8.58 ± 0.15^b^	9.10 ± 0.20^b^
	2008	6.86 ± 0.12^ab^	8.66 ± 0.15^b^	9.17 ± 0.18^b^
	2009	6.80 ± 0.15^b^	8.55 ± 0.30^b^	9.14 ± 0.23^b^
Taezny rubiny	2007	8.14 ± 0.14^c^	9.59 ± 0.13^c^	10.94 ± 0.31^c^
	2008	8.31 ± 0.21^c^	9.79 ± 0.19^c^	11.12 ± 0.26^c^
	2009	8.29 ± 0.22^c^	9.75 ± 0.14^c^	11.02 ± 0.25^c^

Different superscripts in each column indicate the significant differences in the mean at P < 0.05.

Higher antioxidant activity was determined by the ABTS test. In the ´Souzga´ cultivar it was 9.14 grams of AAE/kg FM in a three-year average, in the ´Leningradskaya otbornaya´ cultivar it was 9.94 grams of AAE/kg FM. Antioxidant activity was the highest in the ´Taezny rubiny´ cultivar with a three-year average value of 11.01 grams of AAE/kg FM. In other fruit species, including small berries and stone fruit, the values of antioxidant activity are usually much lower [15]. For example, in cherries values of up to 0.9 grams of AAE/kg FM are seen [22] and in plums the values can reach up to 6 grams of AAE/kg FM [17]. As far as statistical evaluation of the results is concerned, the highest values of the correlation coefficient between antioxidant activity and the total amount of phenolic substances were obtained (in case of the DPPH test r^2^ = 0.88, y = 1.2158x - 3.7383; in case of the ABTS test r^2^ = 0.98, y = 0.7646x - 0.2328). Many authors have noticed a high correlation between TPC and antioxidant activity in fruit [16,23].

The fruit extracts (25%) showed moderate inhibitory ability on nitric oxide (21.89–25.44%), superoxide anion (25.13–28.50%), hydroxyl radical (19.40–23.94%) and lipid peroxidation (11.20–13.90%). European cranberrybush fruit was more effective than other fruit species, e.g., mulberry [24] or apples [25]. ROS are generated in living organisms during metabolism. Excessive amounts of ROS may be harmful because they can initiate biomolecular oxidations which lead to cell injury [26]. ROS are implicated in the pathophysiology of diseases, such as cancer, rheumatoid arthritis, cirrhosis and arteriosclerosis as well as in the degenerative process associated with ageing [27]. Lipid peroxidation is often caused by ROS as an oxidative alteration of polyunsaturated fatty acids. In a biological system, lipid peroxidation generates a number of degradation products and it is found to be an important cause of cell membrane destruction [28].

## 3. Experimental

### 3.1. Description of growing locality

Fruit were harvested in an experimental gene-fund orchard of Mendel University in Brno over the period from 2007–2009. This orchard is situated in the area of the village called Zabcice, approximately 20 km south of Brno, in the Czech Republic. The altitude is 184 m. The average annual temperature and a fifty-year average sum of precipitation are 9 ºC (during the growing season 15.6 ºC) and 553 mm (during the growing season 356 mm), respectively. The soils are classified as gleyed alluvial soils developed on the Holocene calciferous sediments with a marked accumulation of organic compounds. As far as the texture is concerned, the topsoil is loamy and the subsoil clayey-loamy [29].

### 3.2. Sample processing

Fruit were harvested in September from five plants of each cultivar under study within the period of consumption ripeness [7] (thus each year had 5 replicates). Twenty randomly chosen fruits from each tree were used for analyses (*i.e.*, altogether 100 per each cultivar). The cultivars ´Leningradskaya otbornaya´, ´Souzga´ and ´Taezny rubiny´ were used. Their corresponding fruit colours are in case of ´Leningradskaya otbornaya´ and ´Taezny rubiny´ bright red, and as far as the ´Souzga´ cultivar is concerned, its colour is rather dark red.

The fruit of individual trees were processed after the harvest (not later than within two days). Harvested fruits were puréed in a mixer and an averaged sample was obtained by dividing into quarters. Each parameter was measured in five replicates from the fruit taken from each tree of particular cultivars (n = 25). 

### 3.3. Total phenolic content (TPC) and antioxidant activity (TAA) assay

The extraction was performed according to the method described by Kim *et al.* [21], using 10 g of a fresh sample homogenized for 10 seconds in an extraction mixture of hydrochloric acid-methanol-water in a ratio of 2:80:18. Folin-Ciocalteau reagent was used for measurement of TPC. A sample (0.5 mL) was taken and diluted with water in a 50 mL volumetric flask. Thereafter, Folin-Ciocalteau reagent (2.5 mL) and 20-percent solution of sodium carbonate (7.5 mL) were added. The resulting absorbance was measured on a LIBRA S6 spectrophotometer at a wavelength of 765 nm against a blank sample, which was used as reference. The results were expressed as grams of gallic acid (GAE)/kg of fresh mass (FM).

Antioxidant activity was measured using the ABTS (2,2´-azinobis-3-ethylbenzthiazino-6-sulphonic acid) method described by Sulc *et al.* [30]. ABTS (54.9 mg) were dissolved in phosphate buffer (20 mL, pH 7.0; c = 5 mmol/L) and activated to the ABTS^+^ cation radical form by means of an addition of of MnO_2_ (1 g). The resulting solution was intermittently stirred for an activation period of 30 min. Thereafter, the solution was centrifuged for 5 min. and at 7,000 r.p.m. and filtered through a syringe filter (0.25 µm) and a portion of the filtrate (2 mL) was diluted with phosphate buffer to an absorbance of 0.500 ± 0.01, which was measured at the wavelength of 734 nm. After the absorbance was measured, the fruit extract (0.5 mL) was added and the new absorbance value was measured after 20 minutes. 

The DPPH (2,2-diphenyl-1-picrylhydrazyl) assay was done according to the method of Brand-Williams *et al*. [31] with some modifications [32]. The stock solution was prepared by dissolving DPPH (24 mg) in methanol (100 mL) and then stored at −20 °C until needed. The working solution was obtained by mixing the stock solution (10 mL) with methanol (45 mL) to obtain an absorbance of 1.1 ± 0.02 units at 515 nm using the LIBRA S6 spectrophotometer. The fruit extracts (150 µL) were allowed to react with the DPPH solution (2,850 µL) for 1 hour in the dark. Thereafter, the absorbance was taken at 515 nm. The antioxidant activity was calculated as a decrease in the absorbance value using the formula:

Antioxidant activity (%) = (A_0_ – A_1_/A_0_) × 100%

where A_0_ is the absorbance of the control (without the sample) and A_1_ is the absorbance of the mixture containing the sample. The absorbance results were converted using a calibration curve of the standard and expressed in ascorbic acid equivalents (AAE) [33].

### 3.4. Reactive oxygen species scavenging activity assay

For the measurement of reactive oxygen species activity a 25% fruit extract was prepared in phosphate buffer (c = 50 mmol/L, pH 7.0). The hydroxyl radical scavenging activity was assayed according to the method by Ghiselli *et al.* [34]. The extract (1 mL) was mixed with reaction buffer (0.8 mL, KH_2_PO_4_·KOH, c = 0.2 mol/L, pH 7.4; deoxyribose, c = 1.75 µmol/L; iron ammonium sulphate, c = 0.1 µmol/L; and EDTA, c = 0.1 µmol/L). H_2_O_2_ (0.1 mL, c = 0.01 mol/L) was then added to the reaction solution. The solution was incubated for 10 min. at 37 ºC prior to the addition of 1% thiobarbituric acid (0.5 mL) and 2.8% trichloracetic acid (1 mL). The mixture was boiled for 10 min and cooled rapidly. The absorbance of the mixture was measured at 532 nm (LIBRA S6 apparatus).

The nitric oxide scavenging activity assay was done according to the method described by Green *et al.* [35]. The extract (1 mL) was mixed with the reaction solution (1 mL) containing sodium nitroprusside (c = 10 mmol/L) in phosphate buffer (c = 50 mmol/L, pH 7.0). Incubation at 37 ºC for 1 h followed and an aliquot (0.5 mL) was then mixed with Griess’ reagent (0.5 mL). The absorbance at 540 nm was measured.

The superoxide anion scavenging activity assay was done by the method described by Beissenhirtz *et al.* [36] and it is based on the reduction of cytochrome *c*. The extract (1 mL) was mixed with a solution (1 mL) of containing 0.07 units per mL of xanthine oxidase, xanthine (c = 100 µmol/L) and cytochrome *c* (c = 50 µmol/L). After the incubation at 20 ºC for 3 min, the absorbance at 550 nm was determined. 

All tests were performed in triplicate. The scavenging activities of hydroxyl radical, nitric oxide and superoxide anion were calculated as follows:

Scavenging activity (%) = (A_0_ – A_1_/A_0_) × 100%

where A_0_ is the absorbance of the control (without the sample) and A_1_ is the absorbance of the mixture containing the sample.

### 3.5. Lipid peroxidation inhibition activity

The inhibition of lipid peroxidation was assayed by the method by Srivastava *et al.* [37]. Rat liver (5 µg) was homogenized in Tris-HCl buffer (20 mL, c = 40 mmol/L, pH 7.0). The liver homogenate (0.1 mL) was incubated with the sample (0.2 mL of a 25% extract), KCl (0.1 mL, c = 30 mmol/L), FeSO_4_ (0.1 mL, c = 0.16 mmol/l) and ascorbic acid (0.1 mL, c = 0.06 mmol/L) at 37 ºC for 1 h. Thereafter, 1% thiobarbituric acid (TBA, 1 mL) and 15% trichloracetic acid (1 mL) were added. The final solution was heated at 100 ºC in a boiling water bath for 15 min, cooled with ice for 10 min, and then centrifuged at 5,000 r.p.m. for 10 min. The absorbance of the supernatant was measured at 532 nm, using the LIBRA S6 spectrophotometer. The blank was made up by substituting Tris-HCl buffer (c = 50 mmol/L, pH 7.0) for the sample. The inhibition percentage of the formation of TBA-reactive substances was calculated as:

Inhibition activity (%) = (A_0_ – A_1_/A_0_) × 100%

where A_0_ is the absorbance of the control (without the sample) and A_1_ is the absorbance of the mixture containing the sample.

### 3.6. Total flavonoid content assay

The total flavonoid content was determined according to Singleton *et al.* [38]. In a 10 mL Eppendorf tube, the fruit extract (0.3 mL), 30% ethanol (3.4 mL), NaNO_2_ (0.15 mL, c = 0.5 mol/L) and AlCl_3_·6H_2_O (0.15 mL, c = 0.3 mol/L) were added and mixed. After 5 min, NaOH (1 mL, c = 1 mol/L) was added, and the mixture was measured at the wavelength of 506 nm. The total flavonoid concentration was calculated from a calibration curve using rutin as the standard. The results were expressed in g/kg FM.

### 3.7. Ascorbic acid content assay

The determination of ascorbic acid content was carried out by a modification of the method by Miki [39]. The sample (5 g) was extracted in an extraction mixture (methanol-H_2_O-H_3_PO_4_ in the ratio 99:0.5:0.5). The HPLC system used for ascorbic acid analysis consisted of a Supelcosil LC8 chromatographic column (150.0 × 4.6 mm; 5 µm), a Coulochem III electrochemical detector with a guard cell (ESA Inc., Model 5010A, working electrode potential 600 mV and 650 mV), a solvent delivery pump (ESA Inc., Model 582). Chromatographic conditions were: temperature 30 °C, mobile phase a mixture of methanol-H_2_O-H_3_PO_4_(99:0.5:0.5), isocratic elution, flow rate 1.1 mL/min. The retention time of ascorbic acid was 1.9–2.0 min. The content of ascorbic acid was calculated as g·kg^-1^ of FM.

### 3.8. Statistical analysis

The data obtained were analyzed statistically by the analysis of variance (ANOVA) and Tukey´s multiple range test for comparison of means [40]. Correlation functions were calculated using the statistical package Unistat, v. 5.1 and Office Excel® Microsoft. 

## 4. Conclusions

In our measurement, very high contents of polyphenolic substances and ascorbic acid were observed in the fruit of European cranberrybush (*Viburnum opulus* var. *edule* Marsh.). Furthermore, there was a high correlation between these substances and antioxidant activity. From this point of view, the fruit of European cranberrybush have unique properties in comparison with other fruit species. They could become a valuable source of nutritionally important substances for human nutrition. Moreover, they can have a significant influence on strengthening human immunity and the prevention of many diseases. The work deals with cultivar variability, which is described sporadically in literature and new knowledge is gained on ROS and lipid peroxidation scavenging activity of this fruit. In this way the knowledge can support immediate utilization in both theoretical and practical areas – in breeding work, selection, and everyday growing practice. 
